# Safety and efficacy of apatinib combined with iodine-125 in chemotherapy-refractory advanced lung cancer: A case report

**DOI:** 10.1097/MD.0000000000021600

**Published:** 2020-08-14

**Authors:** Yunchao Zhang, Shiqiang Yin, Yingjie Jia, Lei Qin

**Affiliations:** a Department of Oncology, First Teaching Hospital of Tianjin University of Traditional Chinese Medicine; b Administration Department, Sinopharm Group Tianjin Co., Ltd.; c Graduate School, Tianjin University of Traditional Chinese Medicine, Tianjin, China.

**Keywords:** advanced lung cancer, apatinib, iodine-125 radioactive seeds brachytherapy, second-line therapy

## Abstract

**Introduction:**

Apatinib is a novel anti-angiogenic agent that targets vascular endothelial growth factor receptor-2, and is effective in patients with advanced lung cancer who are refractory to first-line chemotherapy. However, there are limited reports on concurrent apatinib therapy with iodine-125 radioactive seeds brachytherapy in elderly patients with advanced lung cancer.

**Patient concerns:**

We describe the first reported case of a 70-year-old woman with advanced lung cancer (T3N3M1, stage IV) who received concurrent apatinib and iodine-125 radioactive seeds brachytherapy after the failure of platinum-based doublet chemotherapy

**Diagnosis:**

The patient was diagnosed with left lower lung cancer with mediastinal lymph node metastasis by chest computed tomography.

**Interventions:**

Initially, apatinib alone was used as second-line cancer therapy. Subsequently, the patient received concurrent apatinib and iodine-125 radioactive seeds brachytherapy.

**Outcomes:**

The patient achieved partial response shortly after undergoing treatment with only apatinib. During the treatment, the tumor continued to respond to apatinib therapy, and the lung metastases were diminished eventually. However, a chest computed tomography scan showed a large cavity in the lung tumor. Thereafter, the patient received concurrent apatinib and iodine-125 radioactive seeds brachytherapy. Unfortunately, she died due to pulmonary infection.

**Conclusion:**

Apatinib alone may be a good second-line therapy for advanced lung cancer patients who are refractory to platinum-based doublet chemotherapy. However, its potential benefits, especially as combination therapy, need further investigation by future prospective clinical studies. Elderly patients with advanced lung cancer may benefit from concurrent apatinib with iodine-125 radioactive seeds brachytherapy when chemotherapy is not tolerated or effective. Further studies are needed to investigate the clinical outcomes and toxicities associated with concurrent apatinib and radiation therapy in patients with advanced lung cancer.

## 1 Introduction

Lung cancer is one of the most fatal malignancies worldwide, with an overall 5-year survival rate of less than 15%. Patients with non-small cell lung cancer (NSCLC), which accounts for more than 85% of all lung cancer cases,^[1]^ are usually diagnosed at an advanced stage.^[2]^ For advanced unresectable NSCLC patients without gene mutations, conventional chemotherapy (platinum-based doublets) and radiotherapy are recommended as the standard first-line treatment. However, for patients who have failed or are intolerant to first-line therapy, a subsequent standard treatment option is still not available, especially for patients who have failed multiple lines of therapy.

Apatinib, an oral small-molecule tyrosine kinase inhibitor targeting vascular endothelial growth factor receptor, has demonstrated a promising anti-tumor effect by inhibiting VEGF-mediated endothelial cell migration and proliferation, as well as by decreasing tumor microvascular density. Apatinib has been clinically used as a third-line treatment in metastatic gastric cancer after approval by the China State Food and Drug Administration. A Phase II clinical study revealed that apatinib demonstrated good efficacy as a third-line treatment for nonsquamous NSCLC.^[3,4]^

Iodine-125 radioactive seeds brachytherapy is an important and effective radiotherapy technique for targeting locally malignant tumors, and is recommended as a standard treatment for early stage prostate cancer by the National Comprehensive Cancer Network Guidelines, American Close Society, Radiation Oncology Society, Urology Society, etc. Furthermore, studies have revealed that this technique is also effective for other malignancies such as lung cancer, rectal cancer, cervical cancer, and liver cancer. Brachytherapy can deliver a continuous low-dose-rate of radiation to tumor lesions directly over a long period of time while sparing the surrounding normal tissues.^[5,6]^ As a result, iodine-125 brachytherapy has been increasingly utilized for the clinical treatment of NSCLC since the 1980s. In recent years, under the guidance of medical imaging techniques, iodine-125 radioactive seeds brachytherapy has been proposed as a strategy to treat medically inoperable tumors.

Until now, few studies have reported the efficacy of apatinib combined with iodine-125 radioactive seeds brachytherapy in patients with NSCLC. In this case study, we have described a patient with advanced lung cancer who experienced excellent tumor response along with acceptable toxicity with this therapy.

## 2 Case presentation

A 65-year-old Chinese woman, was admitted to our hospital on May 6, 2018, with a history of phlegmatic cough with asthma for more than 1 year. Her medical history revealed that she had undergone chest computed tomography (CT) in October 2016, which showed left lower lung cancer and mediastinal lymph node metastasis. A left lung biopsy revealed an adenocarcinoma (moderately differentiated). Neck ultrasound showed a left neck level IV area lymph node enlargement, about 2.2 × 1.7 × 1.4 cm, the door medulla is unclear, consider metastasis. Subsequently, the patient received 8 cycles of first-line chemotherapy consisting of pemetrexed 900 mg combined with carboplatin 60 mg on day 1, repeated every 3 weeks. Synchronous radiotherapy was administrated 33 times to the lung and 25 times to the neck. From June 2017, oral Kemena treatment was administered for nearly 1 month, before discontinuation due to disease progression. From August 2017, the patient received 6 cycles of chemotherapy consisting of paclitaxel 300 mg on day 2 along with bevacizumab 500 mg on day 1. After receiving chemotherapy for a period of 6 weeks, she further received 2 cycles of chemotherapy combined with bevacizumab 500 mg on day 1. However, a chest enhancement CT performed on April 8, 2018, revealed bilateral lung metastases. Additionally, a neck ultrasound revealed enlargement of the left collarbone lymph nodes, the biggest 1 measuring about 2.1 × 1.6 cm.

**Figure 1:**
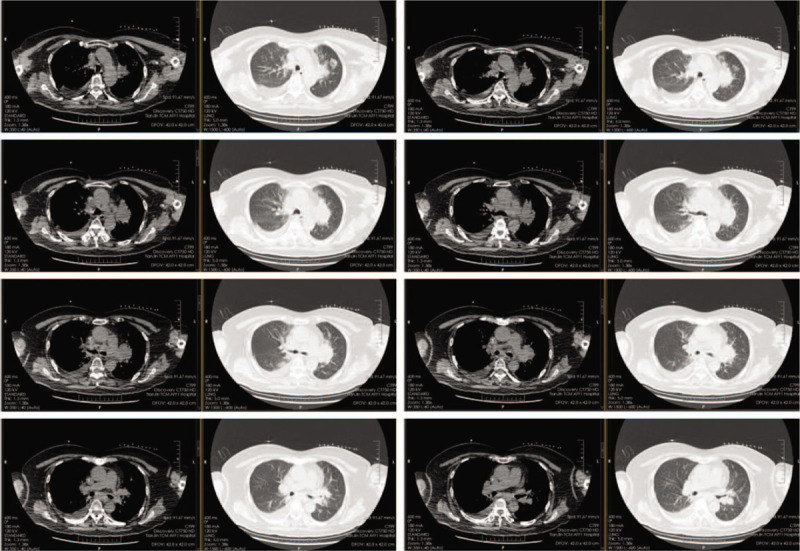
Chest computed tomography before iodine-125 radioactive seeds brachytherapy (May 24, 2018).

**Figure 2:**
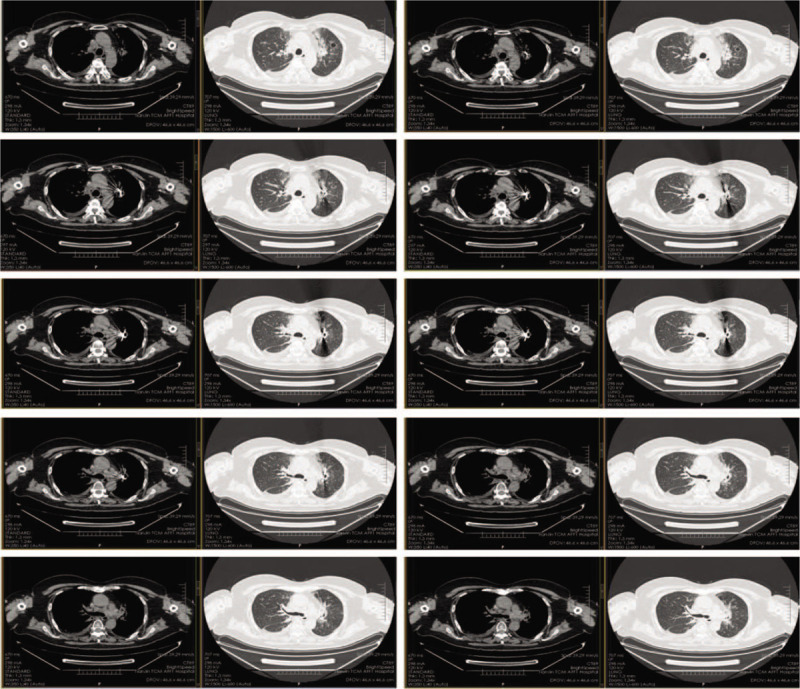
Chest computed tomography after iodine-125 radioactive seeds brachytherapy (June 22, 2018).

On May 24, 2018, the patient received iodine-125 therapy in the left lung with implantation of 30 radioactive particles. All particles were implanted into the tumors under CT guidance. In brief, the patient was placed in a supine position and fixed on the CT bed by a stereotactic body frame. The radioactive particles were implanted after piercing the puncture needle into the CT positioned I 60-layer of the left lung tumor tissue, and the radioactive particles were implanted. A small amount of hemoptysis and cough occurred after the operation, therefore, we gave the patient 90 mL of saline and 1ku of haemocoagulase injection. A week later, the symptoms disappeared and there were no other side effects. At the same time, she received targeted therapy consisting of oral apatinib 500 mg every day. During the therapy, she exhibited high blood pressure, with the highest blood pressure being 167/95 mm Hg, therefore, we gave her nifedipine 30 mg every morning. Blood pressure stablilized after 10 days; the highest was 147/95 mm Hg and the lowest 121/80 mm Hg, and there was no obvious dizziness or headache. One month later, a chest CT revealed a remarkable reduction in the tumour when compared with the pre-therapy chest CT (Fig. 2). On clinical assessment, the Radiation Therapy Oncology Group was grade 1, adverse effects were grade 3, and progression-free surviva was 2 months. However, the patient died due to pulmonary infection 2 months later (Figs. 1 and 2, Tables 1 and 2).

**Table 1:**
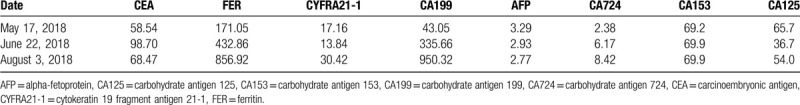
Change of tumor markers.

**Table 2:**
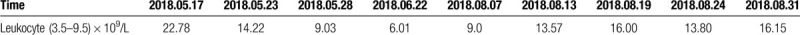
Change of leukocyte.

## 3 Discussion

Lung cancer has the highest cancer morbidity and mortality rates worldwide, and the incidence rate of lung cancer has witnessed an increasing trend in recent years. NSCLC, usually diagnosed at an advanced stage, accounts for more than 70% of all lung cancer cases,^[1]^ and these patients cannot be treated surgically. For NSCLC patients who do not harbor a genetic driver mutation, platinum-based chemotherapy is the standard first-line treatment. However, the efficacy of chemotherapy tends to reach a plateau phase during the therapy period. Anticancer treatment after second-line chemotherapy is indicated if the Eastern Cooperative Oncology Group - Performance Status is satisfactory. However, alternative third or subsequent-line therapeutic regimens are limited, and the efficacy is generally unsatisfactory. Therefore, these patients require newer, safer, and more effective treatments.

Malignant tumors in the middle and late cancer stages are generally associated with poor outcomes. Existing studies have suggested that angiogenesis is a key event during malignant tumor growth and metastasis. Unlike normal blood vessels, tumor blood vessels are commonly characterized by high permeability with irregular morphology. Consequently, normalization of the vasculature by antiangiogenic therapy is an important approach for remodeling the tumor micro environment.^[7]^ In 1971, Folkman was the first to hypothesize the potential therapeutic benefits of targeting tumor angiogenesis. Proteins related to the VEGF family are key regulators of normal as well as tumor angiogenesis and may serve as promising targets for anticancer therapies.^[8]^ In recent years, antiangiogenic therapy has become increasingly popular in cancer treatment and has demonstrated potential benefits.

Apatinib is a novel oral small molecule tyrosine kinase inhibitor developed in China. It has been approved for selectively targeting vascular endothelial growth factor receptor and inhibiting VEGF-mediated vascular endothelial cell migration and proliferation, thereby blocking tumor neovascularization. A Phase III placebo-controlled clinical trial showed that apatinib confers a significant survival advantage over placebo for third-line treatment of gastric cancer.^[3]^ On the basis of this study, apatinib was approved by the China Food and Drug Administration for third-line treatment of advanced gastric cancer. A Phase II placebo-controlled trial investigating the efficacy and safety of apatinib in the third-line treatment of nonsquamous NSCLC, which was first reported at the American Society of Clinical Oncology meeting in 2012 (abstract 7548), indicated that apatinib had promising antitumor activity in advanced NSCLC.^[6]^

Iodine-125 radioactive seeds can be implanted into the tumor permanently and release continuous low-dose X- and γ-rays that provide steady irradiation to the tumor cells at all stages of the cell cycle, with only a negligible radiation dose to the normal tissues adjacent to the lesion^[9]^. It had been shown that iodine -125 seed implantation is a highly effective treatment for patients with primary and secondary tumors of any body system^[9,10]^. The advantages of brachytherapy in the treatment of malignant tumors include:

1) brachytherapy can effectively improve the dose distribution ratio of radiation locally to the cancer tissue;
2) the re-proliferation of tumors is significantly reduced due to continuous radiation exposure;
3) continuous low dose irradiation can cause an increase in the reactive oxygen species level in the anaerobic cells and increase the sensitivity of the tumor cells to rays;
4) fewer toxic side effects;
5) toxic side effects are negligible compared with traditional radiotherapy;
6) implanted iodine -125 particles impart a high dose of radiation to the target area without affecting the surrounding tissues, thus reducing 3the damage to the surrounding normal tissue^[11,12]^.

Iodine-125 brachytherapy alone has been shown to significantly enhance the clinical efficacy and reduce the incidence of myelosuppression compared with chemotherapy.^[13]^ However, iodine-125 brachytherapy may cause some side effects. Although iodine-125 brachytherapy is a minimally invasive treatment and the radioactive seeds are implanted in the tissue permanently to deliver a significant radiation dose inside the tumor, it may also cause a concentrated radioactive exposure to the normal tissue.^[14,15]^ The commonly reported complications of iodine-125 radioactive seeds brachytherapy are numbness, mild pain, and bleeding at the puncture site, infection, postoperative low fever, radiation damage to the surrounding tissues and organs, and particle migration. Radioactive reaction is an inevitable reaction after the implantation of radioactive particles in vivo. Therefore, the most common side effect of radioactive particle implantation therapy is the radiation damage to the adjacent tissues and organs in the local target area after the implantation of radioactive particles. However, as long as dose control is strictly carried out, radiation damage to the normal tissues and organs may be tolerable and reversible. In case of superficial tumors, it may not be possible to completely avoid the occurrence of radiation dermatitis, and it may be helpful to reduce the occurrence of complications by implanting the particles as far into the tumour as possible. Nevertheless, particle migration and even exfoliation occurs from time to time. The key to avoid these complications is an accurate and reasonable preoperative plan, precise implantation, and strict treatment control.

This patient was initially diagnosed with left lung adenocarcinoma following pathological examination and platinum-based doublet chemotherapy was commenced as the first-line therapy. Subsequently, systemic chemotherapy and local radiotherapy in the form of iodine-125 therapy were given to the patient. However, disease recurrence was observed after 2 months.

In this case, apatinib with local radiation as iodine-125 therapy demonstrated good efficacy and safety in the treatment of advanced lung cancer, which indicates that apatinib might be a feasible option for the treatment in advanced lung cancer patients or patients with poor physical condition. Our report may provide some clinical evidence for the future use of apatinib in advanced lung cancer treatment.

Advanced lung cancer may benefit from concurrent apatinib and iodine-125 radioactive seeds brachytherapy when chemotherapy is not tolerated or successful. Further studies are needed to investigate the clinical outcomes and toxicities associated with concurrent apatinib and radiation therapy in advanced lung cancer. Additional data is required to formulate and refine treatment guidelines to maximize treatment benefit and minimize toxicity.

## 4 Conclusion

This case report shows that apatinib with iodine-125 radioactive seeds brachytherapy may provide an alternative for the treatment of patients with second-line treatment failed advanced lung cancer, especially for patients without driver gene mutations for targeted therapy and those with poor performance status. However, the long-term efficacy and patient survival rates need to be confirmed by further research. Larger number of randomized clinical trials are required to determine whether it can be used alone as a first line therapy, or whether it can be used together with other chemotherapeutic agents. We hope the results of this case study will pave the road for successful clinical application of apatinib and its combinatorial therapy.

## Author contributions

**Conceptualization:** Yunchao Zhang, Yingjie Jia.

**Formal analysis:** Shiqiang Yin.

**Investigation:** Yunchao Zhang, Shiqiang Yin.

**Writing – original draft:** Yunchao Zhang, Lei Qin.

**Writing – review & editing:** Shiqiang Yin, Lei Qin.
